# Detection and control of an ongoing international outbreak of hepatitis A among the Irish Traveller community beginning September 2020

**DOI:** 10.1017/S0950268822000309

**Published:** 2022-02-16

**Authors:** David Kelly, Colette O'Hare, Paul McKeown, Fiona Cianci, Sarah Doyle

**Affiliations:** 1 Health Protection Surveillance Centre, HSE, Dublin, Ireland; 2 Department of Public Health, HSE South-East, Kilkenny, Ireland; 3 Department of Public Health, HSE East, Dublin, Ireland

**Keywords:** Community outreach, genomic surveillance, HAV, hepatitis A vaccination, Irish Travellers, marginalised population, outbreak control

## Abstract

Hepatitis A virus (HAV) infection is a notifiable disease in Ireland, with national coverage of clinical and laboratory surveillance. In December 2020, a cluster of 11 HAV cases among the Irish Traveller community was detected. The outbreak investigation identified 61 total HAV cases from September 2020 to November 2021. Sequenced isolates were sub-genotype IA with identical genome sequence. Case-patients were predominantly aged under 18 (77%), hospitalised (46%) and lived on communal residential sites. Mass onsite HAV vaccination was employed following failure of initial ring vaccination to contain the outbreak. This is the largest outbreak of HAV described in Ireland, involving spillover to the UK and Netherlands. We recommend mass HAV vaccination and tailored communication for outbreak control in migratory subpopulations.

## Background

Hepatitis A virus (HAV) infection is statutorily notifiable by clinicians and laboratory directors in Ireland. The incidence of HAV infection reported in Ireland in 2019 was 1.0 per 100 000; 50% less than the European Union (EU) incidence of 2.1 per 100 000 [1]. Due to lower HAV incidence, universal HAV vaccination is not recommended in the national immunisation schedule in Ireland, nor in the majority of EU/European Economic Area states [2].

The Irish Traveller community is an ethnic minority, which accounts for 0.7% of the population in Ireland [3]. Extensive family networks tend to predominate, with social mixing with Roma and other migrant communities, and some inter-regional and international migration of families between Ireland and the UK. A considerable proportion of the Irish Traveller community live in mobile home or caravan accommodation [3], often situated on communal residential sites, with limited access to private sanitation facilities. Members of the Irish Traveller community are generally medically and socially disadvantaged [4], and at an increased risk of infectious diseases [5].

## Outbreak detection

In December 2020, 11 cases of HAV were notified to Departments of Public Health in the East and South-East regions of Ireland over a 3-week period. Nine of the case-patients were members of the Irish Traveller community, living on two separate communal residential sites. The Departments of Public Health HSE East and South-East convened a national multidisciplinary outbreak control team to investigate the cluster.

Our aim is to present the findings of this investigation, to alert other countries of the potential for extended outbreaks of HAV among migratory communities, and to recommend targeted strategies for outbreak prevention and control.

## Case definition

An outbreak case was defined as a confirmed or probable case of HAV infection, with onset of symptoms on or after 1 September 2020.

*Confirmed case*:

- A laboratory confirmed (hepatitis A immunoglobulin M (IgM)) case of HAV infection caused by an isolate with a genome sequence identical to that of the outbreak strain (E62020NOV06) OR
- A laboratory confirmed case of HAV infection caused by an unsequenced isolate AND with an epidemiological link to the Irish Traveller community.

*Probable case*: A clinical case of HAV infection [6], with an epidemiological link to the Irish Traveller community.

## Outbreak investigation

Cases were confirmed by serological detection of hepatitis A IgM. Upon notification, case-patients were interviewed about relevant HAV symptoms and exposures, and asked about links to the Irish Traveller community. HAV isolates underwent genotyping and genome sequencing of the VP1 domain using Sanger sequencing at the National Virus Reference Laboratory.

A clinical surveillance alert was emailed to clinicians in primary care, hospital emergency departments and non-governmental organisations providing health and social care in the East and South East regions, to inform about the hepatitis A outbreak.

A site visit to one communal residential site was conducted in January 2021 by Public Health, Environmental Health, a Traveller community representative and the City Council responsible for sanitation. Drinking-water samples were tested for *Escherichia coli*, total coliforms and chlorination levels. The water drainage, toilets and sewage disposal infrastructure were formally inspected and reported to the municipal authority.

An Epidemic Intelligence Information System (EPIS) alert was issued to all EPIS members and to the UK on 4 February 2021 by the Health Protection Surveillance Centre (HPSC) of Ireland, describing the migratory population affected by the HAV outbreak, and sharing the HAV genome sequence of the outbreak strain.

## Results

**Fig. 1.**
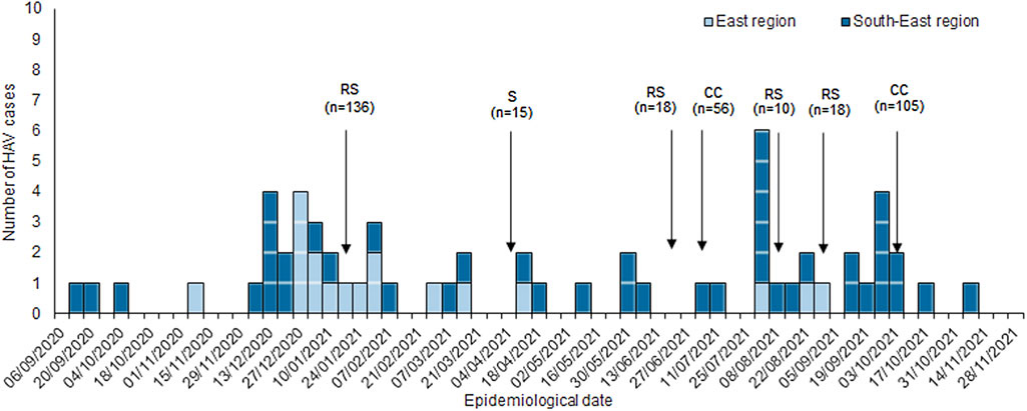
Epidemic curve of all HAV outbreak case-patients by earliest date of onset/diagnosis/notification and region of residence in Ireland, September 2020 to November 2021 (*n* = 61). Residential site (RS), school (S) and community centre (CC) mass HAV vaccination clinics with number (*n*) vaccinated.

From 1 September 2020 to 7 November 2021, a total of 106 HAV cases were notified nationally. Of these, 61 case-patients, all residents of the East and South-East regions of Ireland, met the outbreak case definition (46 confirmed, 15 probable). The initial case-patients arose in the South-East region, had symptom onset in September 2020 and were all members of the Irish Traveller community. Two clusters emerged concurrently in these two regions in December 2020 (Fig. 1), centred on two communal residential sites. A third peak occurred in September 2021, involving an extended family at a social gathering in the South-East region.

Case-patients were predominantly female (61%) and aged under 18 (77%), with 48% identified as living on communal residential sites. Twenty-eight case-patients were hospitalised (46%). No deaths occurred among the 61 case-patients. All case-patients were aged <40 years old (Table 1). Two case-patients had no established epidemiological link to the Irish Traveller community, apart from their local area of residence.

**Table 1.** Descriptive analysis of HAV case-patients linked to the Irish Traveller community outbreak, September 2020 to November 2021 (*n* = 61)

	*n*	%
Age group		
0–9	23	37.7
10–19	26	42.6
20–29	6	9.8
30–39	5	8.2
Unknown	1	1.6
Sex		
Female	37	60.7
Male	24	39.3
Region		
East	18	29.5
South-East	43	70.5
Communal residential site		
Yes	29	47.5
No	32	52.5
Case classification		
Confirmed	46	75.4
Probable	15	24.6
Patient type		
Hospital inpatient	28	45.9
Emergency department	10	16.4
Primary care	8	13.1
Hospital outpatient	1	1.6
Other/unknown	14	23.0
HAV genotyping		
IA	16	26.2
Untypable	7	11.5
No isolate/not typed	38	62.3

No common foodborne vehicle was identified. Drinking-water testing results revealed the absence of coliforms and adequate chlorination levels, and so were not tested for the presence of HAV. Several case-patients confirmed travel history between the East and South-East regions within Ireland, and to and from the UK, through extensive family networks.

On-site inspection of a communal residential site revealed considerable deficiencies in wastewater drainage and sewage disposal infrastructure. Leaking portable toilets, stagnant pools of wastewater, illegal dumping of refuse and overcrowded mobile home units were identified [7].

A total of 16 of 23 HAV isolates yielded successful genotyping results and all were confirmed as HAV sub-genotype IA with identical genome sequence (E62020NOV06), providing a microbiological linkage between the two geographical clusters of Irish Traveller case-patients, as well as with two non-Traveller case-patients in the local areas. In response to the EPIS alert, Public Health England reported a further 27 linked case-patients in the UK, this strain having an identical genome sequence (VRD21_HAV003) to that of the Irish strain. Additionally, a single case-patient was identified in the Netherlands (RIVM-HAV21-049), confirming further international spread of the outbreak. Case-patients in the UK were members of or linked to the Irish Traveller community, with onset dating back to September 2020. No epidemiological link to the case-patient in the Netherlands was identified.

## Outbreak control measures

Case-patients were advised on hygiene and infection prevention precautions. Household contacts were offered HAV vaccination as post-exposure prophylaxis against HAV infection. The clinical surveillance alert was reissued regionally in April 2021, and then nationally in September 2021. In addition, an alert to schools where case-patients had attended during their infectious period was issued.

An Irish Traveller community representative was invited to join the outbreak control team and facilitated community outreach by Public Health. A tailored pictorial information leaflet was produced and disseminated via community representatives and family matriarchs, identified during outreach workshops. Knowledge, attitudes and uptake were gauged to inform and plan vaccination strategy.

The initial ring-vaccination strategy was extended to a mass vaccination strategy of local Irish Traveller communities beginning January 2021. Primary care physicians also provided targeted HAV vaccination to members of the local Irish Traveller community. The rationale for this was threefold: maximisation of vaccine uptake, uncertainty around ascertainment of the exposed population and prolongation of a propagated outbreak.

Free on-site mass vaccination clinics were held January through October 2021 in the East and South-East regions, across schools, communal residential sites and community centres known to local Irish Traveller communities (Fig. 1). A total of 358 members of the Irish Traveller community were vaccinated via these clinics. Uptake of HAV vaccination ranged from 21% (105/500) in local community centres to 76% (136/180) on communal residential sites.

## Discussion

This outbreak underlines the potential for HAV transmission among vulnerable migratory subpopulations across regions and internationally. Transmission occurred primarily among children in this outbreak, which may account for the lack of HAV-related mortality reported to date. The lack of case-patients aged over 40 years was likely explained by immunity to HAV from childhood exposure in the older adult Irish Traveller population, and by the age-demography of the Irish Traveller population in Ireland.

Seroprevalence estimates of HAV antibodies in Belgium, England and Germany estimated that 84–85% of population aged under 30 years were susceptible to HAV infection, compared to 46–50% aged over 30 years [8]. In Ireland, the proportion of the Irish Traveller population aged under 45 years was 84%; much younger compared to the equivalent 63% in the general population [3, 9]. Thus, the vast majority of Irish Travellers are aged under 45, and likely lack immunity to HAV, which may explain the younger age cohort of case-patients.

Transmission was likely facilitated by household contact between families sharing communal residential housing, perpetuated by inadequate access to sanitation facilities and propagated by social mixing across family networks. The high hospitalisation rate suggests under-ascertainment of case-patients, likely due to undetected mild or asymptomatic HAV infection in children. HAV vaccination is not included in the routine childhood immunisation schedules of most EU countries. This, combined with a lack of natural immunity to HAV in younger populations [8], highlights a need for access to free routine HAV vaccination for these vulnerable groups [10]. It has implications for HAV outbreak potential among similar marginalised subpopulations throughout Europe, such as Roma and other migrant communities, who remain vulnerable to HAV transmission because of these risk factors [11–14].

Only two case-patients reported no direct epidemiological link to the Irish Traveller community, indicating minimal spread to the general population. This clustering of case-patients suggests that marginalised subpopulations such as the Irish Traveller community continue to be relatively isolated from the general population. Nevertheless, it highlights the potential for HAV outbreak propagation in countries with low HAV vaccination coverage or little natural immunity to HAV in their population [15, 16]. Both of these case-patients were linked to the outbreak by genome sequencing of their HAV isolates, as were the HAV case-patients from the UK. This serves to highlight the importance of genomic surveillance and sharing of HAV sequences via EpiPulse, as essential for robust investigation and control of HAV outbreaks across Europe [17].

COVID-19 pandemic public health restrictions implemented in January 2021, such as closure of schools, hospitality settings and travel restrictions, may have contributed to reducing HAV transmission. Without these population-level measures, the trajectory of the outbreak may have evolved differently. This also makes the impact of HAV vaccination on control of the outbreak less certain. Nevertheless, the timing of onsite mass vaccination in the East region in January 2021 and South-East region in October 2021 was followed by a sharp decline in HAV cases, and control of the outbreak on affected communal residential sites. The initial ring vaccination strategy of household contacts of case-patients alone proved insufficient to control the outbreak, as demonstrated by successive peaks in cases. Modest (21%) to high (76%) uptake of mass HAV vaccination was achieved via community outreach and mobilisation of Traveller community representatives.

## Conclusion

Tailored communication strategies and outreach were invaluable to empower community leaders with knowledge of, and confidence in HAV vaccination, to promote uptake within the Irish Traveller community. A tailored communication and mass vaccination strategy should be considered for control of similar HAV outbreaks among marginalised subpopulations. Consideration should also be given to the merit of routine vaccination of the wider Irish Traveller community in Ireland, if HAV incidence in this subpopulation remains higher than that in the general population, with resultant harm. The use of EPIS/EpiPulse for sharing of representative sequences can help identify and control clusters of HAV infection in migratory communities more rapidly.

## Data Availability

The datasets used for this study are available from the corresponding author on reasonable request. Restrictions apply to the availability of individual level data, in the interest of patient confidentiality.
